# Emergence of machine language: towards symbolic intelligence with neural networks

**DOI:** 10.1093/nsr/nwad317

**Published:** 2024-01-02

**Authors:** Yuqi Wang, Xu-Yao Zhang, Cheng-Lin Liu, Tieniu Tan, Zhaoxiang Zhang

**Affiliations:** State Key Laboratory of Multimodal Artificial Intelligence Systems, Institute of Automation, Chinese Academy of Sciences, China; State Key Laboratory of Multimodal Artificial Intelligence Systems, Institute of Automation, Chinese Academy of Sciences, China; State Key Laboratory of Multimodal Artificial Intelligence Systems, Institute of Automation, Chinese Academy of Sciences, China; State Key Laboratory of Multimodal Artificial Intelligence Systems, Institute of Automation, Chinese Academy of Sciences, China; State Key Laboratory of Multimodal Artificial Intelligence Systems, Institute of Automation, Chinese Academy of Sciences, China

## Abstract

Inspired by human language, machine language is a novel discrete representation learned from visual data only through playing the speak, guess, and draw game.

Representation learning is a core issue in artificial intelligence (AI). Currently, there exists a disparity in the choice of representation between humans and machines. Humans rely on discrete language for communication and learning, whereas machines utilize continuous features for computation and representation. Discrete symbols are low-dimensional, decoupled and offer robust reasoning abilities, while continuous features are high-dimensional, coupled and possess remarkable abstracting capabilities. In recent years, deep learning [1] has developed the idea of continuous representation to the extreme, using billions of parameters to achieve high accuracies. Although this is reasonable from a statistical perspective, it has other major problems, such as a lack of interpretability, poor generalization and being easily attacked. Both paradigms have strengths and weaknesses, and a better choice is to seek reconciliation.

Inspired by the strengths of human language, we propose a novel approach that combines deep neural networks with symbolic intelligence to create a new form of representation called ‘machine language’. We aim to create a language specifically tailored for machines, combining deep neural networks with symbolic reasoning. Through this fusion, we aim to create a representation that inherits the reasoning abilities of discrete symbols and the abstracting capabilities of continuous features, thereby leveraging the advantages of both paradigms.

Human language is a highly complex and dynamic system that evolves alongside social and cultural changes. Our focus is primarily on the emergence of machine language, specifically from a semantic perspective. Drawing inspiration from the characteristics of human language, we propose three essential properties that machine language should possess. Similar to the early languages found in tribes, which may have been simple in form and grammar, these basic properties are fundamental for the development of a language.

1. *Spontaneous.* The process of language emergence should be spontaneous, resembling the natural evolution of language in early human communities. It should not depend on prior knowledge of human language or require additional data annotations. The development of machine language should be unsupervised or self-supervised [2], occurring through interactions with others and the environment.
2. *Flexible.* The form of machine language should exhibit flexibility, characterized by variable-length discrete symbol sequences. This variability is essential because different individuals may describe the same objects or concepts using varying lengths of language, ranging from concise to elaborate expressions.
3. *Semantic.* A language, in the context of machine language, should possess semantics that can be conveyed through the permutation and combination of basic symbols. It should enable machines to communicate and comprehend information, allowing them to perform specific tasks such as describing objects or providing instructions.

To achieve the aforementioned objectives, a basic idea is to leverage the cooperation among multiple agents to facilitate the automatic learning of a language. This process entails multiple agents working together to solve various tasks within complex environments [3–6]. We begin by simulating this process in the simplest scenario of a two-agent game, aiming to generate a language through their interactions. As depicted in Fig. 1, two agents, speaker A and listener B, engage in a collaborative game. The process of language emergence can be divided into three stages, represented by the three scenes illustrated as follows.

1. *Perception.* Agent A perceives a target image, which in this case is a bird sitting on a tree.
2. *Communication.* Agent A and agent B engage in a communication exchange, utilizing a sequence of symbols to convey information. Agent A, having observed the bird on the tree, attempts to describe what he saw using language. The symbols used in the communication are the targets that both agents aim to learn and understand.
3. *Cooperation.* Agent A and agent B collaborate to solve tasks based on their communication. In this scenario, agent B needs to interpret agent A’s description and identify the bird sitting on the tree based on the communicated symbols. The successful cooperation between the two agents allows them to complete tasks effectively. Specifically, agent B’s tasks involve understanding the language used in communication and making accurate guesses about what agent A is describing. Additionally, agent B needs to visually represent his understanding by drawing the original image.

**Figure 1.**
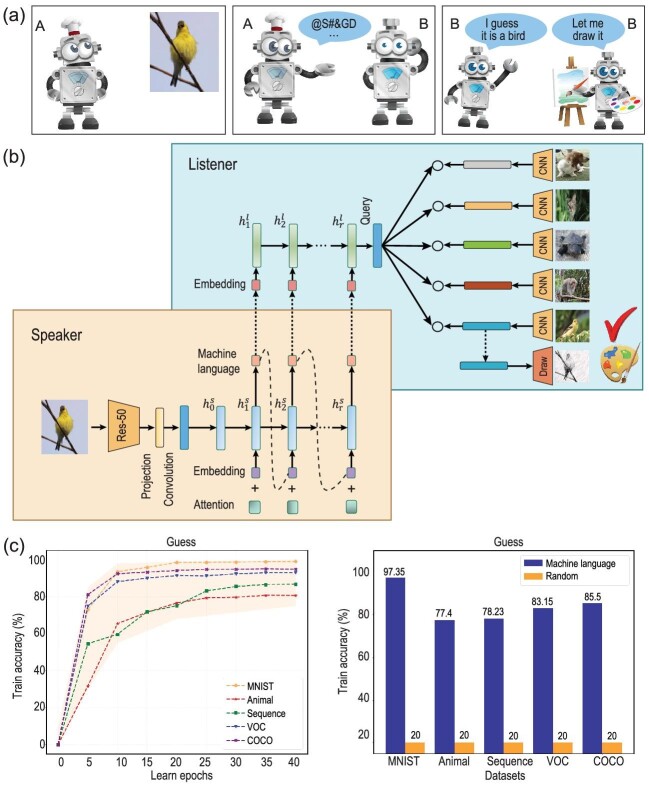
(a) The emergence of language is facilitated through the speak, guess and draw game, depicted from left to right. Given a random image, agent A attempts to describe it using the novel machine language. Agent B, the listener, must comprehend the language and accurately guess what A is describing. Simultaneously, B draws the image based on their understanding. (b) The network structure of the speaker and listener is based on an encoder–decoder architecture. The speaker first perceives an image and generates a sequence of symbols representing the machine language. Subsequently, the listener receives the machine language as input, and outputs a query to make a guess for the correct target within a batch. Additionally, the listener draws the image according to the comprehension of the machine language. (c) To evaluate the effectiveness of our approach, we conduct experiments on five datasets. The left plot illustrates the training accuracy across learning epochs, demonstrating a clear improvement in guessing accuracy with the aid of machine language. The right plot compares the test accuracy compared to random guessing.

Rewards are provided for successful performance in the game, while punishments are given for poor performance. This game is referred to as the speak, guess and draw (SGD) game in this paper. The game can be characterized by a tuple *G* = 〈*D, V, R, A_s_*, *A_l_*, *M*〉, *B* = 〈*T*, …〉. Here, *D* represents the set of all images, *V* denotes the vocabulary restricting the symbols that the agents can use (e.g. 26 characters in English) and *R* defines the range of sequence lengths (e.g. 8–16 symbols). For each turn, a random length *r* ∈ *R* is assigned. Agent A, denoted as *A_s_*, serves as the speaker who observes the target image and generates a variable-length sequence *M* = (*m*_1_, *m*_2_, …, *m_r_*), representing the machine language. Each *m_i_* represents a discrete symbol. Agent B, referred to as *A_l_*, acts as the listener who receives the machine language *M* and decodes the information to solve two tasks: guessing the target image among distractors and drawing the target based on the provided information.

We employ a neural network model to simulate the SGD game process, depicted in Fig. 1. The model follows an encoder–decoder structure, with the speaker acting as the encoder and the listener as the decoder. For a given random image, the speaker first processes it using a convolutional neural network (CNN) to extract the embedded feature. This feature is then fed into a recurrent neural network (RNN) with long short-term memory units to generate a variable-length sequence, representing the machine language to be learned. The listener receives this sequence and processes it using another RNN to produce a feature vector, denoted as query *q*. The game involves three tasks based on this query. The first is *guessing*, where the listener has not seen the target image before and relies solely on the language input from the speaker to make a guess. A batch of images *B* is randomly selected, including distractor images and the target image seen by the speaker. The second task is *drawing*, where the listener, based on their understanding of the language, is asked to draw an image reflecting their interpretation. The drawn image is then compared with the original image, and the reconstruction error defines the loss *L*_draw_. This generative task enhances the semantic understanding of the language. To promote language flexibility, a *regularization* task is introduced. The speaker can describe an image multiple times with different sequence lengths, resulting in different queries *q* from the listener. The consistency of these queries is measured using a regularization loss *L*_regularization_. By incorporating these tasks, the model simulates the collaborative process of speaking, guessing and drawing.

Besides showing the emergence of machine language, we also verified its functionality by comparing discrete language with the continuous feature from three aspects of interpretability, generalization and robustness on diverse datasets and tasks. Discrete language offers inherent interpretability, allowing for the manipulation and modification of semantic meanings. Our experiments demonstrated that discrete language can be purposefully altered to convey different semantic interpretations. Robustness is a crucial characteristic in practical applications, and our experiment evaluated the robustness of discrete language and continuous features. We compared their classification accuracy under different conditions, including the presence of noise and adversarial samples [7]. The results revealed that continuous features suffered a significant decrease in performance when subjected to perturbations; discrete language remained more robust and stable. This can be attributed to the abstract nature of language, which focuses on conveying higher-level semantic information rather than relying on specific visual details. From the generalization perspective, continuous features have shown strong performance in independent and identical distribution settings. However, we argue that the compositionality of language enables better generalization, particularly in out-of-distribution scenarios. While there may be little difference in accuracy between discrete language and continuous features for known categories, language-based representations excel when dealing with new and unknown categories.

In conclusion, the study of machine language represents an exciting and valuable direction in AI research. As AI progresses towards cognitive intelligence, there is a growing interest in integrating symbolic intelligence with neural networks, as advocated by Yoshua Bengio *et al.* in their Turing lecture [8]. Recent advancements, exemplified by models like CLIP [9], DALLE-2 [10] and GPT-4, have demonstrated AI’s potential to learn from cross-modal information, moving beyond traditional methods that rely on human language for learning visual concepts. Our work takes a different perspective, exploring whether machines can develop their own language, known as machine language, through cooperative visual tasks, without relying on human language. By harnessing the potential of visual big data in shaping machine language, we aim to reconcile symbolic intelligence with neural networks, paving the way for more advanced AI systems. This research direction aligns with the transition towards cognitive intelligence and offers a fresh perspective on language learning in AI, going beyond human language-driven approaches. We firmly believe that emphasizing visual information in language emergence can lead to significant progress in AI capability.

## Supplementary Material

nwad317_Supplemental_File
