# Supplementary material for: Emergence of machine language: towards symbolic intelligence with neural networks
Source: Natl Sci Rev. 2024 Jan 2;11(4):nwad317. doi: 10.1093/nsr/nwad317 (PMC10862086; doi:10.1093/nsr/nwad317)
Supplement: nwad317_Supplemental_File [file nwad317_supplemental_file.pdf]

SUPPORTING INFORMATION FOR
Emergence of Machine Language: Towards Symbolic Intelligence with Neural Networks

EXPERIMENTS

Emergence of Machine Language

During the playing of the *SGD* game, we made a fascinating discovery that machines are capable of generating language-like sequences. In each round of communication, we randomly selected a picture from the batch as the target, and the language's vocabulary size was set to 26, with a length range of 8-16 characters. The length of the language was chosen randomly for each round of communication. The detailed results on five datasets are presented in Table 1a. Due to the random selection of target images and sequence lengths for each round, variations in performance can arise from different initial conditions. To mitigate this, we calculated the average results over five repetitions. The experiments unequivocally demonstrated that machines can effectively convey information using discrete symbols. To further evaluate our approach, we compared it with generative models like VAE [1] in Table 1b. To maintain consistency with our experimental setup, we trained generative models during training and utilized the generated latent code during testing to calculate similarity within each batch for the guessing task. The experimental results clearly indicated that the performance of generative models was inferior to our proposed machine language. This advantage can be attributed to the discriminative nature of our *SGD* game compared to the lack of discriminative ability in generative models.

Table 1: Guessing task accuracy of SGD game.

Dataset	Train(%)	Test(%)
MNIST	99.30±0.24	97.35±1.07
Animal	88.57±0.97	77.40±0.88
Sequence	87.65±0.98	78.23±1.13
VOC	94.35±1.59	83.15±1.21
COCO	97.04±0.64	85.50±1.13

(a) Guessing accuracy on diverse datasets

Method	Test(%)
Random	20.00
VAE [1]	51.41±0.92
Machine language	97.35±1.07

(b) Guessing accuracy with generative model

Analysis of Machine Language

In this section, we delve into the fundamental nature of machine language, emphasizing its three key attributes: spontaneity, flexibility, and semantics. The game setting of *SGD* ensures the essential aspect of **spontaneity**. Unlike previous works in communication, we do not rely on human language knowledge [2,3] or utilize additional annotations [4] to facilitate the communication process. The speaker, acting as the encoder, gradually acquires the ability to describe the image using its own language through collaborative play with the listener, serving as the decoder. Regarding **flexibility**, we emphasize two vital aspects that guide our approach. Firstly, we aim to generate variable-length sequences as opposed to fixed-length representations, a common practice in many prior works [3–5]. Secondly, our method embraces diverse descriptions for the same picture. We randomly assign different lengths to the network as conditions, enabling the network to output descriptions of corresponding lengths. Furthermore, we discovered that our approach supports variable-length descriptions under different vocabularies. Different vocabulary sizes (e.g., binary, decimal, alphabet) can lead to distinct machine languages. Remarkably, despite variations in sequence length and vocabulary, the listener can still comprehend the language and make accurate guesses. Tables 2a and 2b showcase results with different vocabularies and variable lengths on the *MNIST* dataset. **Semantics** represents the essence of language, serving as the core differentiator between meaningful language and random sequences. However, exploring the semantics of machine language is challenging as it arises spontaneously. Some prior works [2,6] have attempted to measure the captured semantics in emergent communication protocols, relying on indirect indicators like mean-rank [6]. In contrast, we propose

Table 2: Tables (a) and (b) illustrate the flexibility of machine language. Tables (c) and (d) analyze the machine language from the perspective of semantics.

Vocabulary Size	Train (%)	Test (%)
5	97.72	90.99
10	97.30	91.91
20	98.74	96.18
**26**	**99.50**	**98.60**

(a) Machine language with different vocabulary

Variable Length	Train (%)	Test (%)
4-8	99.10	95.16
8-16	99.34	96.85
**4-16**	**99.50**	**98.60**
16-32	99.14	92.58

(b) Machine language with variable length

Dataset	Train (%)	Test (%)
MNIST	99.10	97.25
Animal	89.80	81.87

(c) Quantitative analysis of semantics

Symbols	Image Features	CR (%)
N * C	gray-scale	0.87
R * I	person	0.98
B * M	sky	0.93
G * M	landscape	0.91
T * F	indoor	0.97
T * G	food	0.80
Y * M	traffic	0.78

(d) Semantic analysis on COCO dataset

a more direct and quantitative method to analyze semantics. By employing a classification task, we aim to measure the performance of distinguishing categories using machine language. After the *SGD* game, the speaker can describe an image using its learned machine language. We utilize machine language as input and the image label as output, training a neural network to establish a mapping. The classification results presented in Table 2c for both the *MNIST* and *Animal* datasets demonstrate the top-1 accuracy of 10 categories. Remarkably, the results in Table 2c affirm that we successfully construct a mapping from machine language to its semantic category. The above-mentioned quantitative analysis relies on datasets with category labels. However, there are a lot of images without labels or specific category labels in real life. To address this limitation, we propose a qualitative analysis method based on language clustering. In our qualitative analysis, we use raw images from the *MS COCO* dataset as an example and perform semantic analysis by comparing the contents of pictures associated with similar machine languages. We observed that the generated machine language often follows a two-stage pattern, with symbols at the beginning and end determining the object features to be described. Fig. 1 illustrates the clustering phenomenon of similar language patterns on the *MS COCO* dataset. Table 2d presents some observed patterns in the *MS COCO* test set. For instance, when the description is $R * I$, the corresponding picture is typically of a person. When the description is $T * G$, it usually corresponds to a picture of food. We summarize features from these images and calculate the Correspondence Rate (CR) to assess the accuracy of the machine's understanding. The results in Table 2d indicate a high correspondence rate.

Discrete Language vs. Continuous Feature

In the robustness experiments, we employ the same backbone for visual feature extraction, where one model produces continuous features, and another generates discrete language representations. After training the encoder, we add a linear layer for classification. The process is similar to current self-supervised learning [7], and we can regard the process of language generation as a proxy task. When Gaussian noise is added, the kernel size is 11, and the sigma is 1.0 and 2.0. When adding salt-pepper noise, the density is 0.1. We used *ZOO* [8] to generate adversarial examples. The experiments are conducted on the *MNIST* dataset. Table 3a presents the comparative results under different noise conditions, averaged over five runs on the test set. In the absence of noise in the dataset (first three rows), the performance of the continuous feature-based representation outperforms the discrete language-based representation. However, when various types of noise, such as Gaussian noise and Salt-Pepper noise, are introduced to the test set, the continuous feature-based representation experiences a sig-

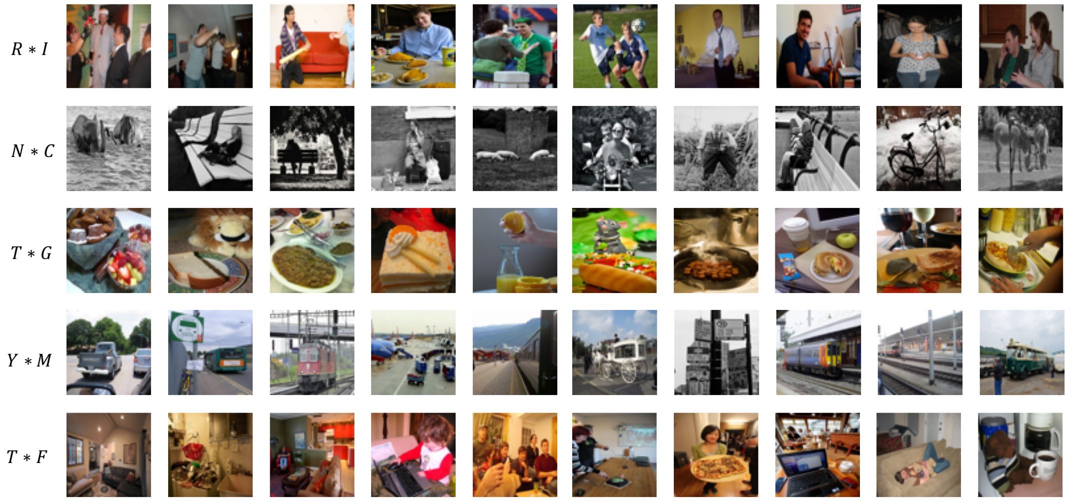

Figure 1: Semantic analysis on *MS COCO*. From the perspective of language, similar languages should have similar visual contents. $R * I$ represents person; $N * C$ represents gray-scale; $T * G$ represents food; $Y * M$ represents traffic transportation; $T * F$ represents indoor scene.

nificant drop in performance. In contrast, the language-based representation exhibits greater stability and robustness. This phenomenon can be understood from the perspective of language's abstract nature, making it more resilient to changes in visual details. Table 3b further illustrates that discrete languages exhibit superior robustness against adversarial samples when compared to continuous features. This finding underscores the advantage of utilizing discrete language representation in the face of adversarial attacks.

Then we make a comparison from the **generalization** perspective. In the context of independent and identical distribution (i.i.d.) settings, continuous features have demonstrated good generalization performance in previous research. However, we propose that language holds the potential for superior generalization performance in out-of-distribution (o.o.d) settings due to its compositionality. This arises from the fact that language can effectively represent complex concepts by combining simple and abstract symbols. To validate this hypothesis, we designed an experiment using the *MNIST* dataset to assess the generalization potential of language in o.o.d. scenarios. Specifically, we divided the numbers from classes 5-9 and 0-4 into two distinct data distributions. For training, we only utilized data from the first five classes (0-4) and evaluated the transfer performance to the new categories (5-9) during testing. Table 3c presents the results of this experiment. For known categories (0-4), there is little difference in accuracy between the discrete language and continuous feature-based representations. However, when it comes to new unknown categories (5-9), the language-based representation outperforms the continuous feature-based representation significantly. This experiment reaffirms the advantage of using language-based representation for o.o.d. generalization tasks, as it has the potential to transfer knowledge more effectively to new and diverse categories.

However, when considering the accuracy and efficiency of discrete symbol representations, it may still be slightly inferior to continuous representations. Due to the auto-regressive output manner, the computational efficiency of this discrete representation may be insufficient. Existing large language models are also auto-regressive, and we believe that more efficient computation will be developed in the future.

DISCUSSION WITH RELATED WORKS

Image Caption

The process of language generation explored in our paper bears similarities to image captioning methods [9,10], which generate textual descriptions from visual images. However, there is a fundamental difference between the two approaches. Image captioning relies on existing human language as supervisory signals, where the goal is to understand the image content from a human perspective and generate descriptive language accordingly. In contrast, the emergence of non-existent language in our work is spontaneous and unsupervised. The primary objective of machine language generation

Table 3: Classification comparison between language and feature

Data	Noise	Language Accuracy(%)	Feature Accuracy(%)
1%	w/o	44.31	**66.25**
5%	w/o	84.33	**89.54**
10%	w/o	94.04	**97.13**
1%	w/ Gaussian	**40.74**	11.30
5%	w/ Gaussian	**78.27**	62.79
10%	w/ Gaussian	**91.36**	84.11
1%	w/ Salt-Pepper	**18.19**	10.19
5%	w/ Salt-Pepper	**45.08**	33.12
10%	w/ Salt-Pepper	**73.69**	59.37

(a) Robustness on different noise: discrete language is more robust than continuous feature when data contain noises.

Adversarial	Language (%)	Feature (%)
w/o	97.66	**99.10**
w/ Adversarial	**68.17**	43.75

(b) Robustness on adversarial examples: discrete language is more robust than continuous feature in front of adversarial attacks.

Class	Language (%)	Feature (%)
0-4 (Known)	99.66	**99.77**
5-9 (Unknown)	**71.72**	63.50

(c) Generalization of new categories: discrete language has stronger o.o.d. generalization than the continuous feature.

is to learn meaningful representations merely from the visual contents, without the need for human annotations. Recently, some works [11–13] have attempted to explore the image captioning task from an unsupervised or semi-supervised approach. However, these methods often involve adjustments to the training process and may still rely on pivot language [11] or additional knowledge from detection and language structure [13] to aid in the captioning task.

Multi-agent Games

Fascinated by the mystery of human language, researchers mimic the emergence process through multi-agent games [5]. Especially in the last five years, simulating language emergence is gaining increasing traction due to the rapid development of deep learning in the studies of language [14] and vision [15]. Given the success of deep learning models in related domains such as image captioning [9] or machine translation [16], research in this space has recently had something of a resurgence with the introduction of models playing referential games. There are two main lines of research in this field. In the first line, emergent language is used as a crucial tool for solving tasks in complex environments. Various tasks have been designed to leverage communication as a means to enhance learning, including navigation [17,18], negotiation [19], translation [20], and more [21]. The second line of work [2–4,6,22–24] is more aligned with our approach, focusing on investigating and analyzing the emergence of communication in referential games. However, previous works have predominantly concentrated on simulating human language rather than exploring novel language emergence. Our proposed machine language stands out as the first approach to generate a non-existent language from the perspective of machines. In contrast to previous works that often use reinforcement learning, we adopt a self-supervised training method. Moreover, the *SGD (Speak, Guess, and Draw)* game setting represents a unique combination of generative and discriminative tasks.

Discrete Representation Learning

Many previous works [25,26] have primarily focused on learning representations with continuous features, while discrete representations offer the promise of better symbolic reasoning and ease of interpretation. Training discrete latent variables in deep learning can be challenging, but auto-regressive models have shown promise in modeling distributions over discrete variables [27]. Recent advance-

ments in vector quantized auto-encoders (VQ-VAE) [28,29] have made significant progress in this direction. However, the difference between our work and VQ-VAE is that our discrete representation is more in line with language characteristics, not only discrete but also variable-length.

IMPLEMENTATION DETAILS

Dataset Preparation

We conducted experiments on diverse datasets, including *MNIST* [30], *Animal* [15], *Sequence*, *Pascal VOC* [31], and *MS COCO* [32].

MNIST: This dataset contains handwritten digits from 0 to 9. The original images were 28×28 pixel grayscale, and we processed them into 64×64 images.

Animal: This dataset contains 10 animal categories selected from *ImageNet*. The input images are 256×256 RGB images.

Sequence: This dataset is a variant of *MNIST*, where we combine multiple digits into a sequence. The sequences can contain two, three, or four numbers, such as 23, 367, and 8907. The resolution of the combined image is 64×64.

Pascal VOC: This dataset is one of the most popular datasets in computer vision, consisting of mostly simple scenes and single objects. We used 33,000 images from *VOC12* as the training set and 9900 images from *VOC07* as the test set.

MS COCO: This dataset is a large dataset that contains images of common objects in real-life scenarios, with mostly complex scenes and multiple objects. We used the original image data without any annotations. The training set and test set are split originally, with 82,000 images in the training set and 40,000 images in the test set.

Model Training

The entire loss function consists of three parts. The first part is the loss for the guessing task, denoted as L_{guess}. We use cross-entropy loss here.

$$L_{guess} = -\sum_{x} p(x) \log q(x) \tag{1}$$

$p(x)$ represents the predicted probability distribution, that is, the probability of predicting a target in a batch. $q(x)$ stands for the real distribution.

The second loss is the reconstruction loss function, denoted as L_{draw}. The primary objective of this loss is to encourage the agent to reconstruct the information of the real target as accurately as possible based on the provided description. This reconstruction process is crucial as it helps the model to refine and improve its language descriptions, leading to more effective communication and better performance in the task.

$$L_{draw} = \begin{cases} 0.5(x_i - y_i)^2, & |x_i - y_i| < 1 \\ |x_i - y_i| - 0.5, & otherwise \end{cases} \tag{2}$$

Our model can only accomplish the painting of the gray-scale image. We used *SmoothL1* loss here. x_i represents the gray value of the pixel drawn at position i, and y_i represents the real pixel value of the target image.

The third loss is the regularization loss, denoted as $L_{regularization}$. Its purpose is to enforce the consistency of descriptions under different lengths. Despite the variation in the length of the descriptions, the information obtained through the decoding process should remain the same. This regularization loss ensures that the model learns to generate compact and informative descriptions that capture essential features regardless of the length of the generated sequence.

$$L_{regularization} = \frac{1}{N} \sum_{i}^{N} (\bar{q} - q_i)^2 \tag{3}$$

Suppose the speaker A_s describes the target picture in N different lengths, q_i represents decoded information under a certain description length. $\bar{q}$ represents the average decoding information.

To train this model, we only need some raw images. The flexibility of the language could be enhanced through regularization. Moreover, the design of our games, i.e., coarsely-grained guessing and fine-grained drawing, promotes the semantic information behind the machine language.

REFERENCES

1. Kingma DP and Welling M. Auto-encoding variational bayes. *arXiv preprint arXiv:1312.6114* 2013; .
2. Havrylov S and Titov I. Emergence of language with multi-agent games: learning to communicate with sequences of symbols. *Neural Information Processing Systems*, volume 30 (2017) .
3. Evtimova K, Drozdov A, Kiela D *et al.* Emergent communication in a multi-modal, multi-step referential game. *International Conference on Learning Representations* (2018) .
4. Lazaridou A, Hermann KM, Tuyls K *et al.* Emergence of linguistic communication from referential games with symbolic and pixel input. *International Conference on Learning Representations* (2018) .
5. Lazaridou A, Peysakhovich A and Baroni M. Multi-agent cooperation and the emergence of (natural) language. *arXiv preprint arXiv:1612.07182* 2016; .
6. Mihai D and Hare J. The emergence of visual semantics through communication games. *arXiv preprint arXiv:2101.10253* 2021; .
7. Liu X, Zhang F, Hou Z *et al.* Self-supervised learning: Generative or contrastive. *IEEE transactions on knowledge and data engineering* 2021; **35**: 857–876.
8. Chen PY, Zhang H, Sharma Y *et al.* Zoo: Zeroth order optimization based black-box attacks to deep neural networks without training substitute models. *Proceedings of the ACM workshop on artificial intelligence and security* (2017) 15–26.
9. Xu K, Ba J, Kiros R *et al.* Show, attend and tell: Neural image caption generation with visual attention. *International conference on machine learning* (2015) 2048–2057.
10. Vinyals O, Toshev A, Bengio S *et al.* Show and tell: A neural image caption generator. *Proceedings of the IEEE/CVF Conference on Computer Vision and Pattern Recognition* (2015) 3156–3164.
11. Gu J, Joty S, Cai J *et al.* Unpaired image captioning by language pivoting. *Proceedings of the European Conference on Computer Vision* (2018) 503–519.
12. Liu X, Li H, Shao J *et al.* Show, tell and discriminate: Image captioning by self-retrieval with partially labeled data. *Proceedings of the European Conference on Computer Vision* (2018) 338–354.
13. Feng Y, Ma L, Liu W *et al.* Unsupervised image captioning. *Proceedings of the IEEE/CVF Conference on Computer Vision and Pattern Recognition* (2019) 4125–4134.
14. Brown T, Mann B, Ryder N *et al.* Language models are few-shot learners. *Neural Information Processing Systems* 2020; **33**: 1877–1901.
15. Deng J, Dong W, Socher R *et al.* Imagenet: A large-scale hierarchical image database. *Proceedings of the IEEE/CVF Conference on Computer Vision and Pattern Recognition* (2009) 248–255.
16. Sutskever I, Vinyals O and Le QV. Sequence to sequence learning with neural networks. *Neural Information Processing Systems*, volume 27 (2014) .
17. Das A, Gervet T, Romoff J *et al.* Tarmac: Targeted multi-agent communication. *International Conference on Machine Learning* (2019) 1538–1546.
18. Jaques N, Lazaridou A, Hughes E *et al.* Social influence as intrinsic motivation for multi-agent deep reinforcement learning. *International Conference on Machine Learning* (2019) 3040–3049.
19. Cao K, Lazaridou A, Lanctot M *et al.* Emergent communication through negotiation. *arXiv preprint arXiv:1804.03980* 2018; .
20. Lee J, Cho K, Weston J *et al.* Emergent translation in multi-agent communication. *International Conference on Learning Representations* (2018) .
21. Mordatch I and Abbeel P. Emergence of grounded compositional language in multi-agent populations. *Proceedings of the AAAI Conference on Artificial Intelligence*, volume 32 (2018) .
22. Andreas J and Klein D. Reasoning about pragmatics with neural listeners and speakers. *Proceedings of the Conference on Empirical Methods in Natural Language Processing* (2016) .
23. Graesser L, Cho K and Kiela D. Emergent linguistic phenomena in multi-agent communication games. *arXiv preprint arXiv:1901.08706* 2019; .
24. Lazaridou A and Baroni M. Emergent multi-agent communication in the deep learning era. *arXiv preprint arXiv:2006.02419* 2020; .
25. Hinton GE and Salakhutdinov RR. Reducing the dimensionality of data with neural networks. *science* 2006; **313**: 504–507.
26. Chen X, Duan Y, Houthooft R *et al.* Infogan: Interpretable representation learning by information maximizing generative adversarial nets. *Neural Information Processing Systems* 2016; **29**.
27. Van Den Oord A, Dieleman S, Zen H *et al.* Wavenet: A generative model for raw audio. *SSW* 2016; **125**: 2.
28. Van Den Oord A, Vinyals O *et al.* Neural discrete representation learning. *Neural Information Processing Systems* 2017; **30**.
29. Razavi A, Van den Oord A and Vinyals O. Generating diverse high-fidelity images with vq-vae-2. *Neural Information Processing Systems* 2019; **32**.
30. LeCun Y, Bottou L, Bengio Y *et al.* Gradient-based learning applied to document recognition. *Proceedings of the IEEE* 1998; **86**: 2278–2324.

31. Everingham M, Van Gool L, Williams CK *et al.* The pascal visual object classes (voc) challenge. *International journal of computer vision* 2010; **88**: 303–338.
32. Lin TY, Maire M, Belongie S *et al.* Microsoft coco: Common objects in context. *Proceedings of the European Conference on Computer Vision* (2014) 740–755.
